# The Association between Emergency Department Super-Utilizer Status and Willingness to Participate in Research

**DOI:** 10.1155/2020/9404293

**Published:** 2020-06-29

**Authors:** Henry W. Young, Emmett T. Martin, Evan Kwiatkowski, J. Adrian Tyndall, Linda B. Cottler

**Affiliations:** ^1^Department of Emergency Medicine, University of Florida, 1329 SW 16th Street, Suite 5270, Gainesville, FL 32610, USA; ^2^Department of Biostatics, University of North Carolina, 135 Dauer Drive, Chapel Hill, N.C. 27599, USA; ^3^Department of Epidemiology, College of Medicine and College of Public Health and Health Professions, University of Florida, 2004 Mowry Road, Room 4218, P.O. Box 100231, Gainesville, FL 32610, USA

## Abstract

**Background:**

Research based on emergency departments (EDs) primarily focuses on medical conditions. There is limited research that investigates patients who willingly participate in research. This current study explored ED super-utilizers' (SUs') and nonsuper-utilizers' (NSUs') attitudes toward research.

**Objective:**

The study assesses the willingness of SUs to participate in research. We hypothesize that the SU population will be as interested as nonutilizers in participating in medical research.

**Methods:**

This prospective observational study stratified participants into SU and NSU cohorts based on their self-reported number of ED visits within 6 months. Surveys were captured in a secured database and analyzed using SAS 9.4.

**Results:**

7,481 completed questionnaires. SUs were more interested in participating in all types of research compared to NSUs. Both groups were most willing to participate in surveys. Neither group was particularly interested in studies that required medications. SUs were not more willing to participate in studies without payment than NSUs. Both groups trusted researchers at the same rates.

**Conclusion:**

Although rarely included in medical research, SUs were more willing to participate in nearly all types of research and expressed a similar trust in medical research when compared to nonsuper-utilizers.

## 1. Introduction

Healthcare spending in the United States is at an all-time high. According to the Centers for Medicare & Medicaid Services (CMS), in 2017, more than $3 trillion dollars were spent on healthcare services in the United States. This was more than twice the amount spent in 2010 and represented 17.9% of the nation's gross domestic product [1]. The reason for rising healthcare costs is multifaceted and includes expansion of health insurance coverage under the Affordable Care Act and an increase in residential mental health and substance abuse facilities [2]. Studies have found that a large amount of healthcare resources are utilized by a small percentage of individuals [3]. The Agency for Healthcare Research and Quality found that 1% of patients in the United States were responsible for 21% of the entire healthcare spending in 2010 [4]. Due to rising healthcare costs, there has been a renewed national focus on reducing healthcare expenditures by developing and evaluating interventions and strategies targeted toward individuals who utilize healthcare services at higher rates than the general population [5–8].

Super-utilizers (SUs) are defined as individuals in healthcare who utilize a disproportionate amount of healthcare resources [4]. Among Medicaid patients, SUs were found to have more hospital stays, longer length of stays, and higher hospital costs per stay [1, 4]. The 30-day readmission rate was nearly 9 times higher for SUs than nonsuper-utilizers (NSUs). In the emergency department, SUs represent approximately 4.5% of all emergency department patients, but account for 28% of all emergency department visits [4]. This can be in part that SUs have more comorbidities and healthcare needs than NSUs [4, 9].

It has been stated in the past that this disproportionate utilization of resources may be due to unmet health needs and lack of access to appropriate settings. Due to the potential impact that SUs have on healthcare expenditures, continued research focused on this population is needed for more appropriate allocation of healthcare resources.

Very few studies evaluate the SU population outside the hospital from a community perspective, and no studies have assessed the interests of ED SUs to participate in research studies. The current analysis assesses the willingness of SUs in the community to participate in research, evaluate what type of research they would be interested in, and evaluate their trust in medical researchers. We hypothesize that the SU population will be as interested as nonutilizers in participating in medical research and trust in medical researchers would be no different than NSUs.

## 2. Methods

For this study, individuals were approached in community settings in North-Central and Northeast Florida through a community engagement program, HealthStreet. HealthStreet utilizes the community health worker (CHW) model to assess community members' health concerns and needs. CHWs approached individuals at public gathering places such as libraries, shopping centers, and community centers. Interested individuals were assessed with a University of Florida Institutional Review Board approved informed consent and a Health Intake Assessment. Within the Health Intake Assessment, individuals were asked about previous research experience, their interest in research, and their willingness to participate in research. Individuals were asked their willingness to participate in different types of studies including: surveys, studies requiring an overnight stay, studies that require providing blood samples including for genetic testing, and studies involving use of medical equipment. A commonly used definition of super-utilizers in the medical literature is an individual that averages 4 ED visits per year [4]. Individuals that reported being evaluated in the emergency department 2 or more times over the last 6 months—which averages 4 visits per year—were identified as super-utilizers in this study. Data for this analysis were collected from October 2011 through December 2015. Group differences were analyzed using the chi-squared test for categorical variables and *t*-test for continuous variables. SAS 9.4 was used to analyze the data.

## 3. Results

Though CHWs contacted 9,134 individuals, informed consent was completed for 7,481. Among these individuals, CHWs interviewed 6,839 community members yielding a participation rate of 75%. Approximately, 14% of the individuals included in the study met the criteria to be classified as SUs for a total of 962 individuals.

As seen in Table 1, SUs were more commonly female with an average age just over 40 years. SUs vs. NSUs did not differ by race or Latino/Hispanic ethnicity but did differ by marital status with SUs being more likely to be divorced, separated, or widowed (36% vs. 29%; *p* < 0.0001).

SUs, compared to NSUs, were also more likely to be less educated, unemployed, have a higher average household size (3.3 vs. 3.1; *p* = 0.03), and report food insecurity (65% vs. 43%; *p* < 0.0001). Importantly, SUs vs. NSUs were more likely to be uninsured (45.5% vs. 40.1%; *p* < 0.0001) or have Medicaid or Medicare insurance (39.2% vs. 36.1%; *p* < 0.0001). The top health concerns were different between groups with the SUs being more concerned with conditions causing muscle and bone problems, weight problems, cancer, heart problems, and mental health conditions (see Table 2).

All participants were asked about their perceptions of medical research. Groups did not differ in prior participation in research; however, SUs were significantly more likely to be interested in participating in research (97% vs. 92%; *p* < 0.0001) than NSUs.

Participants were assessed on their interests in specific study types including surveys, studies that only involved review of their medical records, studies that involved acquiring a blood sample, studies that involved genetic testing, studies that involved taking a medication, and those that involved staying overnight in the hospital or involved the use of medical equipment.

Overall, SUs were more interested in participating in all types of research compared to NSUs. Both groups were most willing to participate in surveys and least willing to volunteer in studies that required them to take medications. Although SUs were also more likely to say they would participate in every type of study, their willingness to participate in unpaid studies was the same as NSUs (79.6% vs. 77.5%; *p* = 0.1453). Both groups trusted researchers and research at the same rates (see Table 3).

## 4. Discussion

To our knowledge, this is the first study of this size to assess ED SUs' interests in medical research. As we hypothesized, super-utilizers were not less trusting of medical researchers or medical research and more willing to participate in medical research than NSUs. This represents a large cohort of individuals that the literature has shown which is very dynamic and with more comorbidities than others who are interested and willing to participate in research despite few ever being involved in research [10, 11]. This population also has unique health concerns that due to their low representation in medical research in the past may not be fully explored if they are not included in research.

Researching SUs presents a unique opportunity to learn more about individuals who utilize a disproportionate amount of healthcare resources. When compared to similar developed countries, the United States spends more of its gross domestic product on healthcare-related expenditures than any other [4]. As health expenditures continue to increase at rates that are not sustainable, more research is needed to understand the needs of this population which will allow better care and decrease the need for unnecessary visits.

Health care disparities are defined by Healthy People 2020 as a particular type of health difference that is closely linked with social, economic, or environmental disadvantage [12]. In this study, the SUs had a greater rate of being food insecure, unemployed, less educated, and uninsured. Studies have demonstrated that individuals that are economically disadvantaged face barriers in accessing healthcare, present with more advanced disease, receive poorer quality of care, and are more likely to die of the disease and have worse health outcomes for acute and chronic conditions [13–16]. Multiple studies have demonstrated that insurance expansion associated with the Affordable Care Act has been associated with conflicting impacts on emergency department utilization among uninsured [17, 18]. Although the degree to which these factors impact emergency department utilization is unclear, it does define this population as a vulnerable population. Due to these differences, SUs are at increased risk of suffering from health disparities which may lead to loss of opportunities to be in clinical studies. If SUs were more involved in medical research, then advancements could be made to reduce the need for these frequent emergency department visits while still meeting the needs of this vulnerable population.

The SU population includes individuals commonly underrepresented in medical research and thought to be difficult to recruit and retain in research [19, 20]. Commonly cited barriers for both enrollment and retention in clinical trials include transportation, lack of access to primary care physicians, delayed diagnosis, multiple comorbidities which may exclude them from studies, and lack of appropriate childcare. Some studies suggest that due to difficulty in recruitment and retention of this population in biomedical research, studies may be disproportionately excluding this population [21, 22]. Similar to prior research, our study demonstrated that this cohort of SUs is as interested in participating in research as NSUs despite these barriers [23, 24].

Trust in research and medical researchers has been identified as a primary determinant in research participation [25–27]. A common cause of mistrust in research is perception of exploitation or negative past experiences with healthcare which is particularly high among racial and ethnic minorities [28]. Though mistrust is commonly identified as a barrier to research participation, trust in medical research and researchers is often cited as a significant facilitator for research participation [25–28]. Similarly, our sample of ED SUs was found to have high trust in medical research and willingness to participate in research. As this population represents an important component of the healthcare population, the addition of the super-utilizer population to medical research will assist with the generalizability of clinical studies and promote greater diversity in research participants.

The social factors among our ED SUs make this population economically disadvantaged and more susceptible to unethical practices within research and according to the “common rule” a vulnerable population. Compensation of individuals for their participation in research is a common practice that has been associated with increased recruitment rate and increased willingness to participate in research [29]. However, if payment for research is excessive relative to the risks of the study or what the individual is asked to do, then it can be coercive and unethical [30]. Despite these social factors, super-utilizers were not less willing to participate in research when payment was not provided, suggesting financial motivations are not the sole motivator for participation in research in this population.

Among our study population, SUs were found to have different health concerns than NSUs. In our cohort, SUs were more concerned about mental health, cardiovascular disease, and muscle or bone problems than NSUs. These conditions are all significant causes of morbidity and mortality in the US and are responsible for significant economic and societal burden. Among these diagnosis, significant health disparities exist including significant unmet need, poorer quality of care, and disparate healthcare utilization within these populations [31–33].

Limitations of the study include self-reported information which may suffer from recall bias regarding ED visits and conditions. However, the recall period was brief and the incident referred to a significant event (ED visits) that a person is likely to remember. Strengths of the study include a large diverse sample that was not restricted to one healthcare system.

## 5. Conclusion

Although rarely included in medical research, super-utilizers were more willing to participate in nearly all types of research and expressed a similar trust in medical research when compared to nonsuper-utilizers. Future research should consider the use of qualitative interviews to explore other factors that may contribute to SUs' attitude and willingness to participate in research and potential biases of researchers. Data from this research can assist in the development of targeted interventions to increase SUs' participation in research.

## Figures and Tables

**Table 1:** Demographics by emergency department utilization.

	Super-utilizers (*N* = 962)	Nonsuper-utilizers (*N* = 5,877)	*p* value
Gender			
Female	63.7%	56.2%	<0.0001
Male	36.3%	43.8%	
Average age			
Female	40.5	43.3	0.0001
Male	45.9	42.8	0.0005
Race			
Asian	0.2%	1.1%	0.0603
African American	62.3%	62.6%	
Caucasian	30.7%	29.7%	
Other	6.9%	6.5%	
Latino/hispanic^*∗*^	4.6%	5.2%	0.4311
Marital status			
Never married	49.1%	49.7%	<0.0001
Married	15.0%	21.1%	
Divorced/separated/widowed	36.0%	29.3%	
12+ years of education	71.4%	79.8%	<0.0001
Currently employed	21.6%	36.8%	<0.0001
Veteran status	10.0%	10.0%	0.9683
Household size (including participant)	3.3	3.1	0.0384
Food insecure (not enough $ to buy food)	64.7%	43.4%	<0.0001
Insurance status			
Private insurance	14.9%	28.8%	<0.0001
Medicaid/medicare	39.6%	31.2%	
Uninsured	45.5%	40.1%	

**Table 2:** Top 8 health concerns^†^ by emergency department utilization.

	Super-utilizers (*N* = 923) (%)	Nonsuper-utilizers (*N* = 5,330) (%)	*p* value
Hypertension	30.6	31.1	0.7539
Diabetes	20.5	21.7	0.3870
Muscle and bone problems	25.2	18.2	<0.0001
Dental problems	16.8	16.6	0.8756
Weight problems	12.4	16.4	0.0019
Cancer	11.6	15.7	0.0012
Heart problems	16.1	13.2	0.0180
Mental health	15.2	10.7	<0.0001

^†^Among those with at least one health concern.

**Table 3:** Research perceptions by emergency department utilization.

	Super-utilizers (*N* = 962)	Nonsuper-utilizers (*N* = 5,877)	*p* value
Ever been in a health research study	16.4%	17.6%	0.3387
Interested in participating in research	97.2%	92.1%	<0.0001
Would you volunteer for a health research study…			
That only asked questions about your health?	95.0%	92.9%	0.0168
If researchers wanted to see your medical records?	91.0%	85.3%	<0.0001
If you had to give a blood sample?	90.1%	83.8%	<0.0001
If you were asked to give a sample for genetic studies?	87.0%	83.2%	0.0033
If you might have to take medicine?	71.2%	59.8%	<0.0001
If you were asked to stay overnight in a hospital or clinic?	82.3%	70.2%	<0.0001
If you might have to use medical equipment?	87.9%	81.1%	<0.0001
Would you participate in a study if you did not get paid?	79.6%	77.5%	0.1453
Average trust in research (1–10 scale, 1 = “not at all,” 10 = completely)	7.5	7.2	0.0624
Average trust in researchers (1–10 scale, 1 = “not at all,” 10 = completely)	7.4	7.2	0.1573

## Data Availability

Data for this manuscript are available upon request.
